# Establishment and validation of a nomogram clinical prediction model for osteoporosis in senile patients with type 2 diabetes mellitus

**DOI:** 10.1038/s41598-024-56127-w

**Published:** 2024-03-04

**Authors:** Jing Li, Xiaolong Zhou, Jing Wen, Shiping Liu, Xingfu Fan

**Affiliations:** 1 Department of General Medicine, Affiliated Hospital of North Sichuan Medical College, Nanchong, 637000 China; 2 Department of Emergency, Affiliated Hospital of North Sichuan Medical College, Nanchong, 637000 China

**Keywords:** Type 2 diabetes mellitus, Osteoporosis, GNRI, Risk factors, Endocrine system and metabolic diseases, Diabetes, Metabolic bone disease

## Abstract

This study aimed to develop a predictive nomogram model to estimate the odds of osteoporosis (OP) in elderly patients with type 2 diabetes mellitus (T2DM) and validate its prediction efficiency. The hospitalized elderly patients with T2DM from the Affiliated Hospital of North Sichuan Medical University between July 2022 and March 2023 were included in this study. We sorted them into the model group and the validation group with a ratio of 7:3 randomly. The selection operator regression (LASSO) algorithm was utilized to select the optimal matching factors, which were then included in a multifactorial forward stepwise logistic regression to determine independent influencing factors and develop a nomogram. The discrimination, accuracy, and clinical efficacy of the nomogram model were analyzed utilizing the receiver operating characteristic (ROC) curve, calibration curve, and clinical decision curve analysis (DCA). A total of 379 study participants were included in this study. Gender (OR = 8.801, 95% CI 4.695–16.499), Geriatric Nutritional Risk Index (GNRI) < 98 (OR = 4.698, 95% CI 2.416–9.135), serum calcium (Ca) (OR = 0.023, 95% CI 0.003–0.154), glycated hemoglobin (HbA1c) (OR = 1.181, 95% CI 1.055–1.322), duration of diabetes (OR = 1.076, 95% CI 1.034–1.119), and serum creatinine (SCr) (OR = 0.984, 95% CI 0.975–0.993) were identified as independent influencing factors for DOP occurrence in the elderly. The area under the curve (AUC) of the nomogram model was 0.844 (95% CI 0.797–0.89) in the modeling group and 0.878 (95% CI 0.814–0.942) in the validation group. The nomogram clinical prediction model was well generalized and had moderate predictive value (AUC > 0.7), better calibration, and better clinical benefit. The nomogram model established in this study has good discrimination and accuracy, allowing for intuitive and individualized analysis of the risk of DOP occurrence in elderly individuals. It can identify high-risk populations and facilitate the development of effective preventive measures.

## Introduction

Type 2 diabetes (T2DM) is the most common type of diabetes (DM), accounting for about 90% of all diabetes cases worldwide^1^. As life expectancy increases, the incidence of diabetes also rises with an aging population. The International Diabetes Federation predicts that the absolute number of people with diabetes will grow by 46% by 2045, particularly in middle-income countries such as China, where the growth will be extremely rapid^2^. Osteoporosis (OP), on the other hand, is a skeletal disease characterized by decreased bone mass, destruction of bone tissue microstructure, and increased bone fragility, leading to pathological fractures^3^. Currently, the estimated global prevalence of OP is 19.7%, as reported in a review by Xiao^4^, but there are slight variations between countries and regions, Salari reported the highest prevalence of OP in older Asian adults, at 24.3%^5^.

In recent years, there has been growing concern about the relationship between diabetes and osteoporosis, resulting in the concept of diabetic osteoporosis (DOP). DOP is a chronic bone disease caused by long-term exposure to high glucose increasing the risk of fractures and osteonecrosis in diabetes patients, eventually leading to fragility fractures and trabecular deterioration^6^. Now, DOP is considered to be one of the major complications of DM^7^. A meta-analysis revealed that the prevalence of OP among patients with T2DM in China was 37.8%. Notably, the prevalence was higher in women compared to men, and it demonstrated an age-related increase. Among the elderly population aged over 60 years, the prevalence reached 40.1%^8^. Due to decreased bone mineral density (BMD) and increased bone fragility in the elderly, the occurrence of DOP can have severe consequences. Fractures caused by DOP have become one of the leading causes of disability and death in the elderly. The causes of DOP are not yet fully understood, and there are currently no definitive clinical measures to prevent it. Therefore, it is important to identify the risk of DOP and screen the high-risk groups.

The present study aims to establish a nomogram by identifying the risk factors of DOP, which may provide a recommendation for prevention and treatment.

## Methods

### Participants

**Figure 1:**
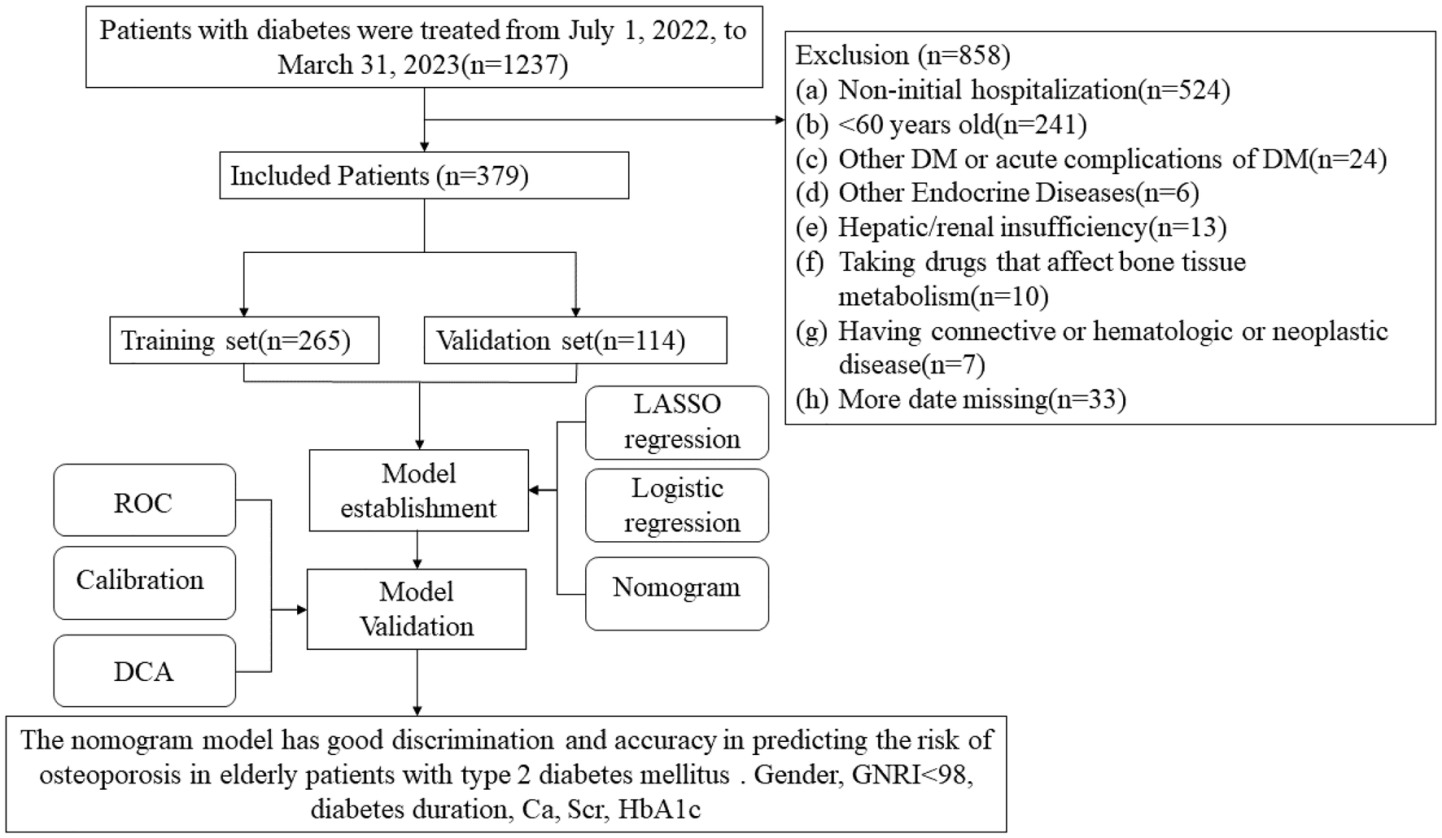
Research pathway diagram.

The present study included patients diagnosed with T2DM who were admitted to the Affiliated Hospital of North Sichuan Medical College between July 2022 and March 2023. All data were obtained from the hospital’s database. The research flow diagram is presented in Fig. 1. This study was approved by the Ethics Committee on Biomedical Research, Affiliated Hospital of North Sichuan Medical College (approval number: 2023ER200-1). As this study is a single-center retrospective study, the review committee waived the requirement for written informed consent. Patient confidential data was removed from the entire dataset before analysis. The study was conducted in accordance with the Declaration of Helsinki.s

The inclusion criteria are as follows: (1) age ≥ 60 years; (2) T2DM diagnosed following the DM diagnostic criteria of the World Health Organization in 1999^9^; (3) BMD measured by dual-energy X-ray absorptiometry (DXA). The exclusion criteria are as follows: (1) other types of DM, acute complications of DM including Diabetes ketoacidosis and hypertonic hyperglycemic syndrome; (2) other endocrine diseases such as hyperthyroidism, hyperparathyroidism, etc.; (3) patients with liver and/or renal insufficiency; (4) patients who had been taking drugs affecting bone metabolism in the past six months, such as calcium, vitamin D and glucocorticosteroid; (5) patients with connective tissue diseases, hematologic diseases, malignant tumors and other serious chronic diseases; (6) patients with incomplete important data or missing indicators of target variables.

The research subjects were divided into modeling and validation groups according to 7:3 random sampling using the R package and classified into T2DM and DOP groups according to BMD results.

### Data collection

After conducting a literature review and analyzing the interaction between glucose metabolism and bone^10,11^, we have identified 33 potential risk factors that may be associated with DOP in patients with type 2 diabetes. These factors include (1) general information: age, gender, body mass index (BMI), blood pressure, smoking history, alcohol consumption, diabetes duration, diabetes family history, and insulin use history; (2) the occurrence of chronic complications in T2DM: diabetes mellitus with poor glucose control, diabetic nephropathy, diabetic retinopathy, diabetic peripheral neuropathy, diabetic peripheral vasculopathy, diabetic foot;(3)hematological indicators: white blood cells (WBC), platelets (PLT), hemoglobin (Hb), serum calcium (Ca), serum phosphorus (P), uric acid (UA), blood creatinine (SCr), fasting blood glucose (FBG), glycated hemoglobin (HbA1c), triglycerides (TG), total cholesterol (TC), high-density lipoprotein (HDL-C), low-density lipoprotein (LDL-C), alkaline phosphatase (ALP), and serum albumin (ALB). The GNRI values were calculated according to the formula, GNRI = 1.489 × serum albumin (g/L) + 41.7 × admission weight (kg)/ideal weight (kg);ideal weight was calculated according to the following formula: men = height (cm) − 100 − [(height − 150)/4], women = height (cm) − 100 − [(height − 150)/2.5]; Based on previous reports in the literature, we classified GNRI ≥ 98 as no nutritional risk and GNRI < 98 as nutritional risk according to the GNRI values^12^.

### Statistical analysis

The statistical analysis of this study was carried out mainly through R software (version 4.2.1) and SPSS software (IBM, version 26.0). The continuous variables of this study were described by mean and standard deviation (SD), and the statistical significance of the differences between two groups was analyzed by the Student’s *t*-test. Normality continuous variables were described by median [*M*(*P*_*25*_*,P*_*75*_)], and the statistical significance of the differences between two groups was analyzed by the Mann–Whitney U-test. Categorical variables were described by frequencies and percentages, and the statistical significance of the differences between the two groups was analyzed using the chi-square test. The least absolute shrinkage and selection operator (LASSO) and multivariate logistic regression analyses were performed to explore the risk factors for elderly DOP, and the rms program package was used to draw the nomogram. The clinical prediction model was internally validated using the Bootstrap resampling method with B = 1000 repetitions, and perform validation set for external validation. The ROC, AUC, calibration curve and DCA were used to assess the discrimination and predictive value of the nomogram prediction model, which was graded as (1) 0.5 < AUC ≤ 0.7, low predictive value, (2) 0.7 < AUC ≤ 0.9, moderate predictive value, and (3) 0.9 < AUC < 1, high predictive value. The grading of the concordance index (C-index) was equivalent to the AUC value. In this study, p < 0.05 was considered to be statistically significant.

## Results

### Demographics

The study included 379 elderly patients with type 2 diabetes, of which 178 (46.97%) were male with an average age of 70. The incidence of OP in elderly patients with T2DM was 35.1% (133/379). A random split was performed using the R package with a 7:3 ratio resulting in 265 cases for the training group and 114 cases for the validation group. The demographic data and the comparison between the groups are shown in Table 1. Briefly, smoking history, alcohol consumption, and diabetic nephropathy showed statistically significant differences ( p< 0.05), the remaining 30 factors are effective in validating the nomogram prediction model that was developed from the training group for the follow-up study.

**Table 1:** Demographics and baseline data.

Items	Training group (n = 265)	Validation group (n = 114)	*t/*(*Z*)*/*[*x*^*2*^ ] value	*p value*
Gender/case (%)			[3.191]	0.074
1 (Male)	116 (43.8)	62 (54.4)		
2 (Female)	149 (56.2)	52 (45.6)		
Age (years)	70 (65, 75)	69.9 (64, 75.8)	(− 0.592)	0.554
BMI	23.8 (21.1, 25.9)	23.9 (21.5, 25.7)	(− 0.224)	0.822
Systolic blood pressure (mmHg)	133 (133, 133)	133 (133, 133)	(− 1.454)	0.146
Diastolic blood pressure (mmHg)	80.2 ± 11.1	79.1 ± 11.2	− 0.852	0.395
Smoking history/case (%)	64 (24.2)	41 (36.0)	[4.98]	0.026
Alcohol consumption,/case (%)	31 (11.7)	29 (25.4)	[10.286]	0.001
Family history of diabetes/case (%)	21 (7.92)	9 (7.89)	[0]	1.000
Diabetes duration (years)	10 (5.0, 16.0)	10 (5.25, 15.0)	(− 0.023)	0.982
Insulin use history /case (%)	101 (38.1)	43 (37.7)	[0]	1.000
Diabetes with poor glycemic control/case (%)	230 (86.8)	101 (88.6)	[0.1]	0.752
Diabetic nephropathy/case (%)	94 (35.5)	55 (48.2)	[4.929]	0.026
Diabetic retinopathy/case (%)	133 (50.2)	61 (53.5)	[0.231]	0.631
Diabetic peripheral neuropathy/case (%)	156 (58.9)	75 (65.8)	[1.327]	0.249
Diabetic peripheral vasculopathy/case (%)	165 (62.3)	73 (64.0)	[0.045]	0.833
Diabetic foot/case (%)	21 (7.92)	9 (7.89)	[0]	1.000
WBC (× 10^9^ /L)	6.51 (5.35, 8.08)	6.97 (5.34, 8.24)	(− 0.527)	0.599
PLT (× 10^9^ /L)	188 (147, 230)	185 (140, 234)	(− 0.197)	0.844
Hb (g/L)	128 (116, 137)	129 (116, 142)	(− 0.933)	0.351
Ca (mmol/L)	2.22 (2.11, 2.34)	2.26 (2.15, 2.35)	(− 1.398)	0.162
P (mmol/L)	1.03 (0.90, 1.13)	1.04 (0.92, 1.19)	(− 0.843)	0.399
UA (μmol/L)	302 (239, 367)	311 (250, 382)	(− 1.059)	0.290
SCr (μmol/L)	69.9 (57.0, 86.8)	74.0 (57.9, 97.1)	(− 1.436)	0.151
FBG (mmol/L)	10.2 (7.79, 13.5)	10.1 (7.70, 13.4)	(− 0.001)	1.000
HbA1c (%)	9.45 (7.82, 11.4)	9.71 (8.34, 11.8)	(− 1.203)	0.229
TG (mmol/L)	1.52 (1.13, 2.42)	1.54 (1.15, 2.90)	(− 0.931)	0.352
TC (mmol/L)	4.61 (3.72, 5.45)	4.77 (3.74, 5.40)	(− 0.395)	0.693
LDL (mmol/L)	2.57 (1.95, 3.31)	2.55 (1.95, 3.29)	(− 0.097)	0.923
HDL (mmol/L)	1.19 (0.97, 1.45)	1.16 (0.96, 1.38)	(− 0.805)	0.421
ALP (U/L)	85.0 (68.0, 104)	85.5 (68.0, 103)	(− 0.03)	0.976
ALB (g/L)	42.1 (39.7, 44.3)	42.0 (39.0, 44.5)	(− 0.008)	0.994
GNRI	107 (98.6, 114)	107 (99.1, 114)	(− 0.129)	0.897
GNRI stratification/case (%)			[0.134]	0.714
0 (GNRI ≥ 98)	62 (23.4)	24 (21.1)		
1 (GNRI < 98)	203 (76.6)	90 (78.9)		

### Univariate variable screening and multifactorial logistic regression analysis of DOP occurrence in the elderly

**Figure 2:**
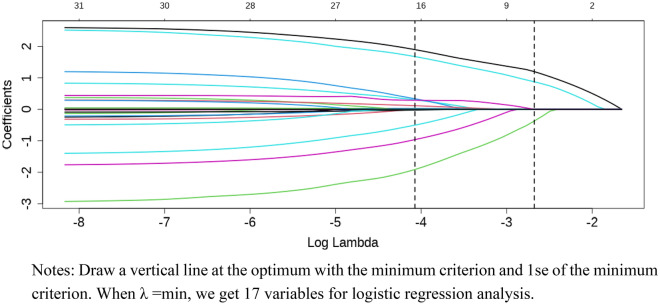
LASSO regression model cross-validation plot.

**Figure 3:**
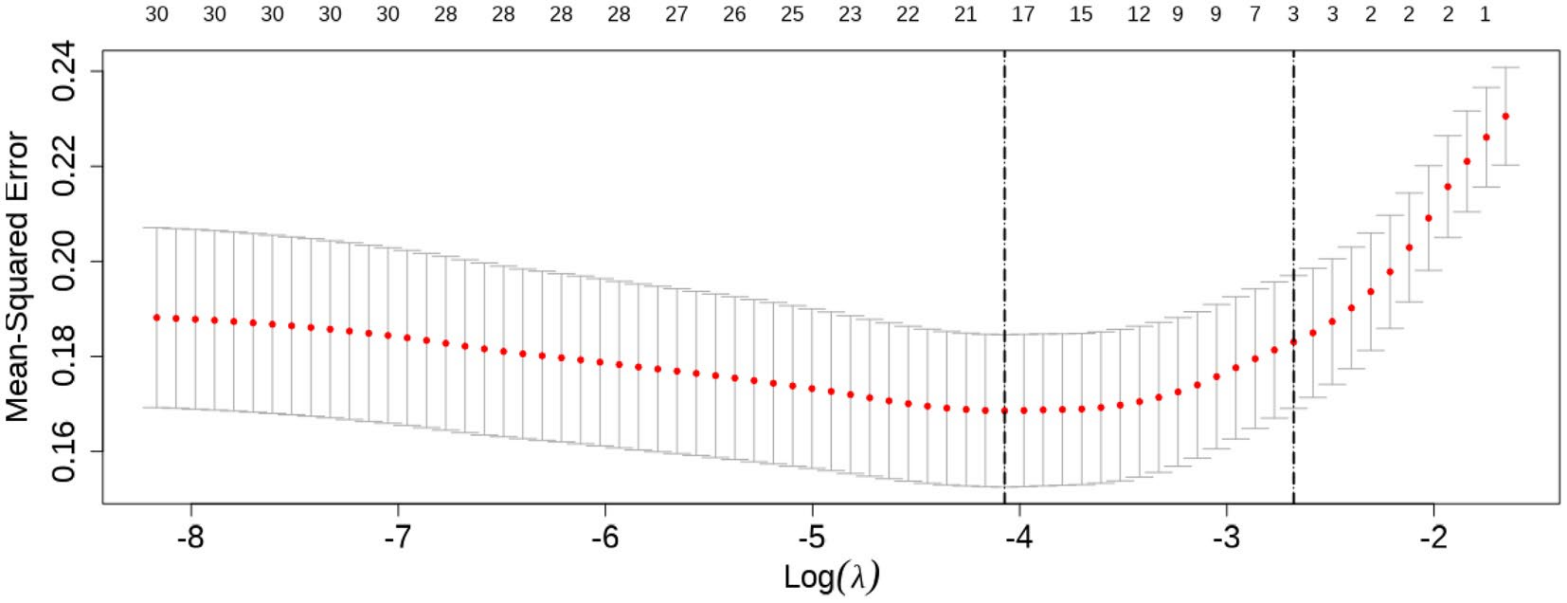
Coefficient profile plot of predictors.

Thirty independent variables were analyzed to determine their correlation with DOP. The LASSO regression was used to reduce the number of independent variables, and ten-fold cross-validation was conducted to select the most relevant variables based on lambda.min. Through this process, 17 predictors were identified. The pathway of variable shrinkage can be viewed in Fig. 2, while the results of cross-validation are presented in Fig. 3. After performing multivariate logistic regression analysis, the following variables showed statistical significance: Gender (OR = 8.801, 95% CI 4.695–16.499,  *p*< 0.001), GNRI < 98 (OR = 4.698, 95% CI 2.416–9.135, p< 0.001), Ca (OR = 0.023, 95% CI 0.003–0.154, p< 0.001), HbA1c (OR = 1.181, 95% CI 1.055–1.322, *p* = 0.004), duration of diabetes (OR = 1.076, 95% CI 1.034–1.119,* p* < 0.001), and SCr (OR = 0.984, 95% CI 0.975–0.993,  p= 0.001), as shown in Table 2.

**Table 2:** Multivariate logistic analysis based on training groups.

Subjects	B value	SE value	*Wald χ*^2^ values	p value	OR value	95% CI
Females	2.175	0.321	46.009	< 0.001	8.801	4.695–16.499
GNRI < 98	1.547	0.339	20.798	< 0.001	4.698	2.416–9.135
Diabetes duration (years)	0.073	0.020	13.228	< 0.001	1.076	1.034–1.119
Ca (mmol/L)	− 3.789	0.979	14.071	< 0.001	0.023	0.003–0.154
HbA1c (%)	0.166	0.058	8.318	0.004	1.181	1.055–1.322
SCr (μmol/L)	− 0.016	0.005	11.152	0.001	0.984	0.975–0.993
Constant	4.779	2.424	3.889	0.049	118.997	–

### Creation of a nomogram for predicting the risk of DOP occurrence in the elderly

**Figure 4:**
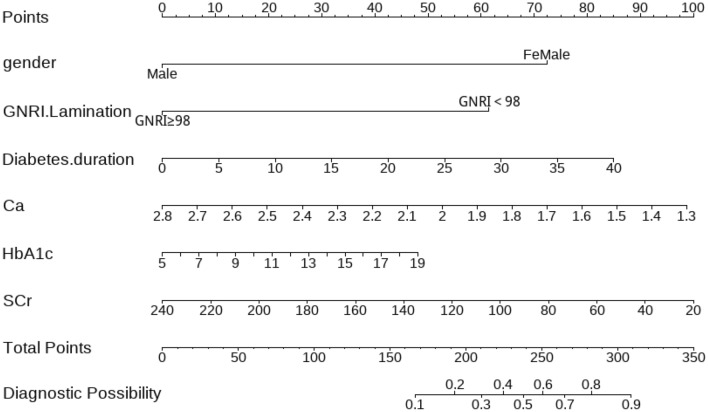
The nomogram of the DOP occurrence in the elderly.

As per the findings of multivariate logistic regression, a nomogram was developed to predict the odds of OP in elderly patients with T2DM. In personalized healthcare, the specific values of gender, GNRI < 98, Ca, HbA1c, duration of diabetes and SCr can be used to determine the corresponding points on the nomogram. The Fig. 4 represents this diagram, where the scores of each independent predictor are on the upper scale, and the total scores of the six factors for each case of subjects are on the lower scale. The total score corresponds to the Diagnostic Possibility at the bottom of the diagram, which shows the subject's risk of DOP occurrence. Taking the patients in our study as an example, consider an elderly female patient with nutritional risk (GNRI < 98) had a diabetes duration of 12 years, a blood Ca of 2.03 mmol/L, an SCr of 36.5 μmol/L, and an HbA1c of 13.11%, the total score of 331.5 calculated from our nomogram showed a prevalence of DOP greater than 90%. Indeed, this patient was a DOP patient. In the contrast, when evaluating an elderly male patient with no nutritional risk (GNRI > 98) had a 10-year history of diabetes, a blood Ca of 2.36 mmol/L, an SCr of 88.3 μmol/L, and an HbA1c of 7.82%, the total score calculated from our column chart is 131, which does not yet reach the minimum value of our nomogram risk score, suggesting that this patient has a very low risk developing DOP.In fact, this patient was only a simple T2DM patient. Hence, the predictive performance of our nomogram model is still acceptable.

### Validation of the nomogram

**Figure 5:**
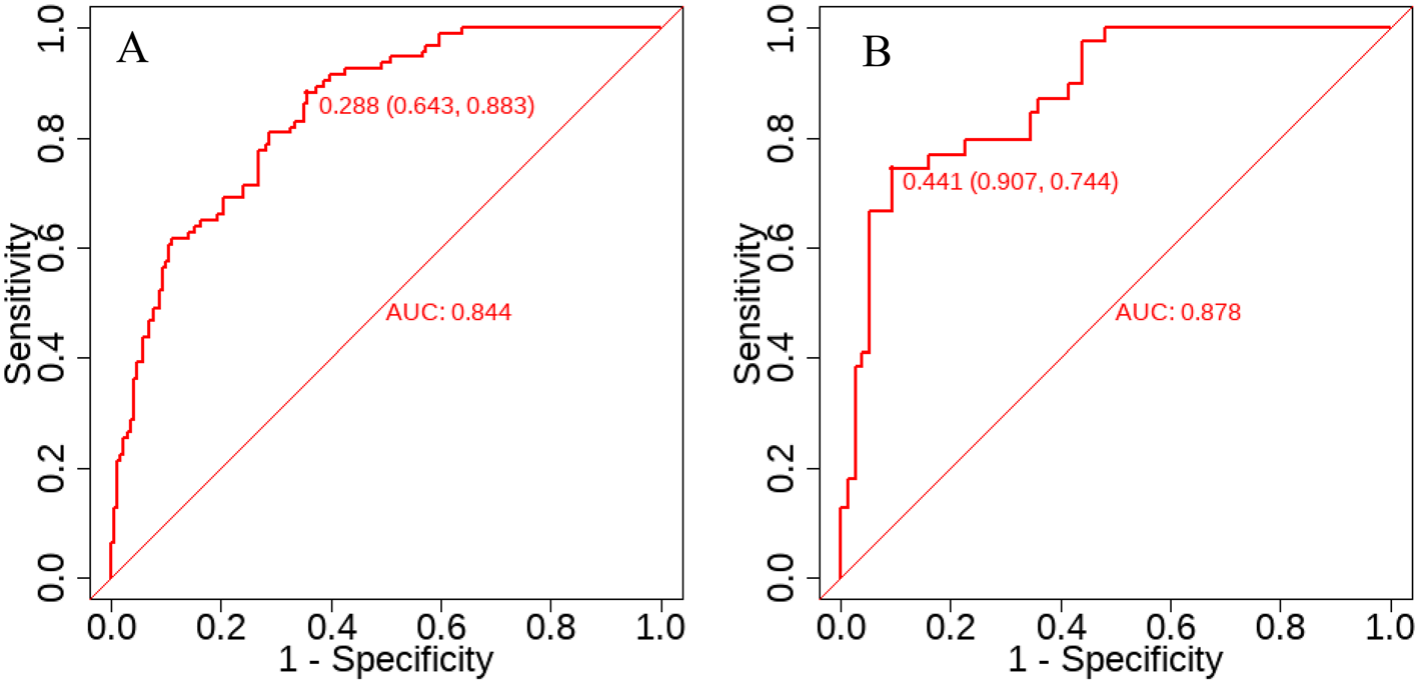
ROC curves of nomogram based on the data of the training set (**A**) and validation set (**B**).

**Figure 6:**
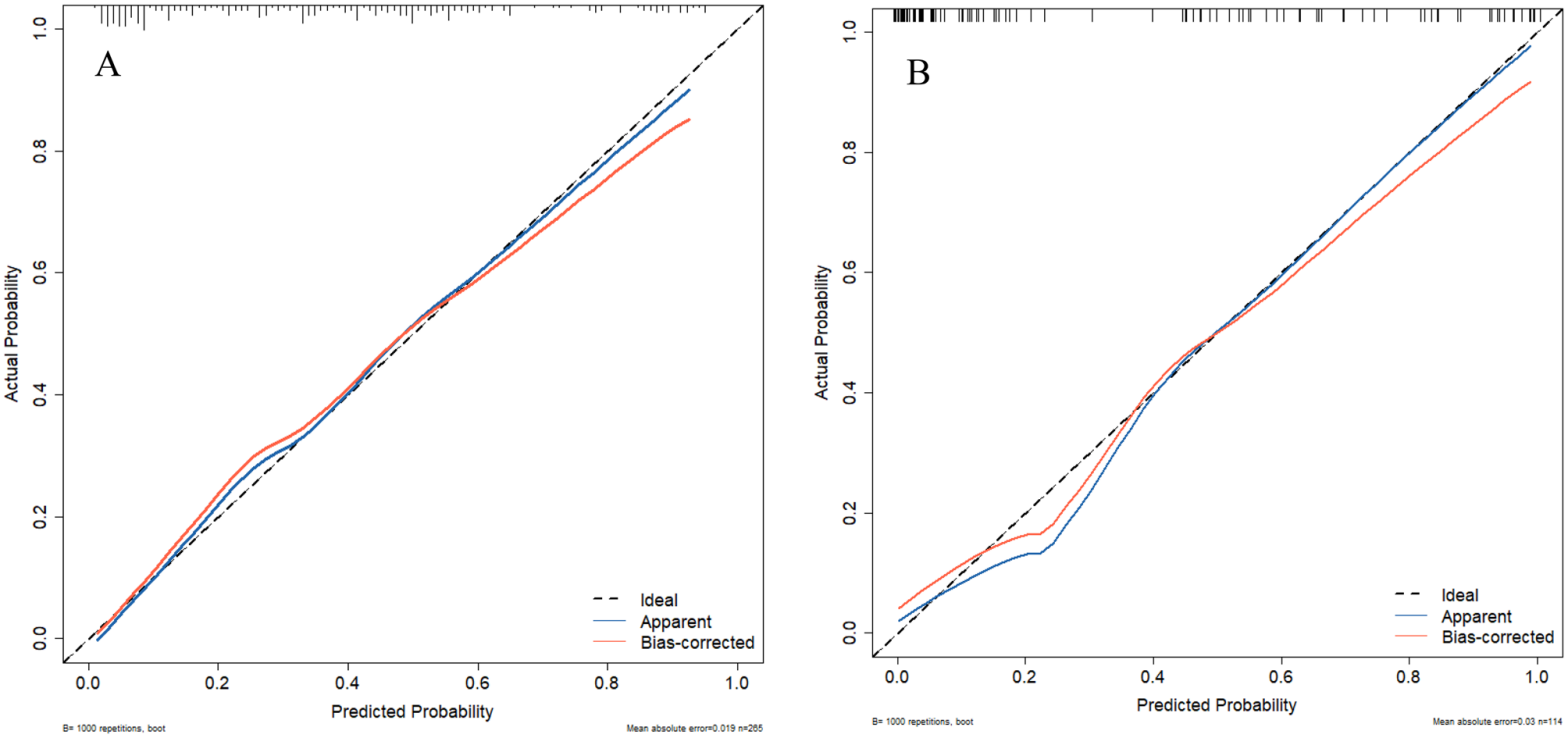
Calibration curve of the nomogram based on the data of training set (**A**) and validation set (**B**).

To assess the validity of the predictive model, ROC curves were generated for both the training and validation sets. Figures 5 and 6 displays the ROC curves and calibration curves for the training and validation groups. Of which Fig. 5A shows the training group, the prediction performance of the nomogram model displayed a high predictive ability, with an AUC of 0.844 (95% CI 0.797–0.89, cutoff value = 0.288, sensitivity = 0.883, specificity = 0.643), while Fig. 5B shows the validation group, the prediction performance of this nomogram model also displayed a high predictive ability, with an AUC of 0.878 (95% CI 0.814–0.942, cut-off value = 0.441, sensitivity = 0.744, specificity = 0.907). The findings indicate that the clinical prediction model for the DOP nomogram developed in this study has a moderate predictive value (AUC > 0.7) and a good discriminatory ability. Afterwards, using the Bootstrap resampling method with 1000 repetitions, calibration curves were created for the training and validation sets. The Apparent and Bias-corrected lines show that the Brier score was 0.154 with a slope of 1 and P = 0.872 in the training sets (Fig. 6A). In the validation sets, the Brier score was 0.14, the slope was 1.16, and P = 0.918 (Fig. 6B). These results indicate good consistency between the model's predicted probabilities and the actual occurrence probabilities, suggesting that the nomogram clinical prediction model for DOP in this study had a certain degree of calibration.

**Figure 7:**
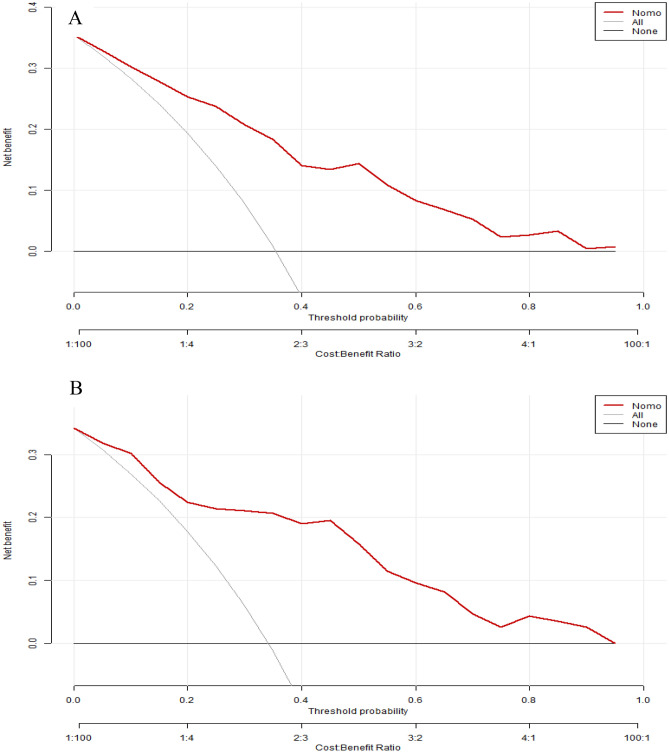
Decision curve analysis curve of the nomogram based on the data of training set (**A**) and validation set (**B**).

To assess the clinical utility of the model, DCA curves were plotted. The DCA curves demonstrate that the net benefits for both the training and validation sets are significantly higher than the two extremes, indicating good clinical value, as depicted in Fig. 7A,B. Moreover,the loss-to-gain ratio remained consistently less than 1, indicating good clinical efficacy of the model.

## Discussion

The Asia Pacific Consortium on Osteoporosis (APCO) raise the most commonly risk factors for osteoporosis were: excessive alcohol consumption, a family history of osteoporosis and/or fractures, smoking, low BMI, age over 70 years, and early menopause. And the laboratory tests that were commonly recommended in the guidelines included calcium, alkaline phosphatase, 25-hydroxyvitamin D and creatinine et al.^13^. In this study, we retrospectively analyzed the mentioned above clinical data and laboratory indicators of 379 cases of elderly T2DM, and included readily available laboratory and anthropological indicators, e.g., gender, GNRI, duration of diabetes mellitus, HbA1c, blood Ca, blood SCr. By a series of statistical methods, We identified gender, GNRI < 98, Ca, HbA1c, diabetes duration and SCr as independent influencing factors for DOP in T2DM patients. We developed a simple and accurate nomogram, which was internally validated, shows that the model has good efficacy and clinical applicability.

It has been shown that T2DM is associated with poor bone health and that DOP as a complication of T2DM leads to an increased risk of fracture, which in turn worsens the prognosis of DOP and increases mortality^14^. The gold standard for osteoporosis diagnosis is BMD^15^, DOP is a systemic metabolic bone disease with increased fracture risk due to decreased bone mass or destruction of the bone microstructure complicating DM, which cannot be detected by dual-energy X-ray in the early stages^16^. However, BMD measurement by DXA is easily affected by the soft tissue and bone morphology of the measurement site^17^. The results of DXA may vary from one site to another and require high equipment, which may lead to a delay in the diagnosis of DOP in primary care units due to a lack of resources. Therefore, the development of simple and practical personalized early prediction tools is essential and clinically relevant. Although there are currently many screening tools for OP, their applicability and effectiveness remain challenging. There are studies that show the sensitivity of osteoporosis self-assessment tool for Asians (OSTA) in identifying subjects with suboptimal bone health was only 32.3%, with an AUR of only 61.8%^18^. In another study, the researchers compare of the discrimination and calibration of the Fracture Risk Assessment Tool (FRAX) and Garvan fracture risk calculator for predicting fractures in postmenopausal women aged 50–64 at baseline. The results showed that the specificity and the sensitivity of both tools for detecting incident hip fracture during 10 years of follow-up was low: Garvan was 30.6% and 16.0%, FRAX was 43.1% and 59.2%^19^. The study by Tan et al. also development a risk prediction model for OP in elderly patients with T2DM, their predictive model based on seven predictors: age, sex, hypertension, coronary heart disease, cerebral infarction, hyperlipidemia, and past surgical history, which lacks of laboratory indicators, and it could identify underlying OP, with an AUC of 0.713, specificity of 65.5%, and sensitivity of 67.5%^20^.In addition, their nomogram predicts DOP in elderly patients with a probability of only 0.05–0.45.

In this study, gender, GNRI < 98, Ca, HbA1c, diabetes duration, and SCr were found to be independent influences for the development of DOP. In older women, ovarian function decreases and the level of estrogen in the body decreases abruptly, which is involved in molecular signaling in bone through activation of the estrogen receptor^10^. The lower level of estrogen in older women could increase bone resorption and reduce the ability to absorb calcium and phosphorus, and osteoclasts proliferate, leading to bone loss, which is more prone to DOP and even fracture^21^. Older adults are one of the most heterogeneous and vulnerable groups, and they face a higher risk of nutritional problems^22^.The prevalence of malnutrition in geriatric hospitalized patients has been estimated to range from 30 to 60%^23^. Therefore, in this study we also included the index GNRI, which reflects nutritional status. In this study, GNRI < 98 (with nutritional risk) was the second largest influence weight after gender, which is an independent risk factor for the development of DOP in the elderly, this is basically consistent with Wang et al.^24^, who found that GNRI was negatively correlated with T2DM suffering from OP. Moreover, compared with BMI, ALB, and age, GNRI is a more powerful predictor of OP complicating T2DM. It has also been shown that high GNRI (with no nutritional risk) is an independent protective factor for OP in elderly T2DM patients, and it is a stronger predictor of OP in elderly T2DM patients than nutritional scores such as COUNT and PNI^25^. GNRI is used to assess the nutritional status and risk of malnutrition in elderly patients^26^. It takes into account serum albumin levels and body weight, providing a comprehensive indicator of nutritional status^27^. Studies have shown that lower GNRI scores are associated with increased risk of DOP. GNRI < 98 is a risk factor for the development of DOP, which may be explained by hypo-proteinemia through NF-κB factor and other inflammatory cytokines activate osteoclasts and inhibit osteoblast^28^. Moreover, hypoproteinemia and low body mass index lead to decreased synthesis of insulin-like growth factor-1 (IGF-1) in patients with T2DM, which inhibits osteoblast proliferation, leading to decreased osteoblast numbers, prolonged osteoclast lifespan, increased bone resorption, and decreased bone remodeling^29^. Lean patients have decreased skeletal machine loading, which is detrimental to bone formation, making them more susceptible to DOP.

In this study, we found that HbA1c and diabetes duration were independent risk factors for DOP, and Ca and SCr were the protective factors for DOP. Increased HbA1c indicated that patients had poor glycemic control in the last 3 months, and the longer diabetes duration indicated that glucose metabolism was abnormal for a longer period. The prolonged hyperglycemia in the body could induce apoptosis of osteoclasts and inhibit their differentiation through oxidative stress, formation of end products of advanced glycosylation and other pathways, causing reduced osteogenic capacity of bone tissue, changes in bone structure, bone loss, and triggering OP^30,31^. In addition, high glucose will lead to osmotic diuresis, blocking the reabsorption of calcium and phosphorus in renal tubules, and resulting in the loss of calcium, phosphorus, and many other trace elements via urine^32^. As diabetic patients get older, their organs function less efficiently. This results in a decrease in the body's ability to absorb calcium. The imbalance of calcium in the body affects bone density and metabolism, leading to bone loss and increasing the risk of developing DOP^33^. SCr is a metabolite of phosphocreatine in human muscle, which depends on the total mass of skeletal muscle and is mainly excreted by glomerular filtration and excreted in urine, which is easy to assess and measure, However, SCr may easily affected by age, gender, and protein intake. Some studies have shown that it can be used as a surrogate marker for skeletal muscle mass when protein intake is adequate^34^. In case of normal renal function, its increased value represents higher muscle mass, and muscle and bone can interact with each other through biodynamic effects, nutritional status of the organism, and hormonal changes in endocrine secretion^35^, and higher muscle mass can prevent the development of DOP. In this study, SCr was negatively correlated with concomitant DOP in elderly patients with T2DM, suggesting that SCr is a protective factor for elderly patients with DOP, which is in line with the study of Guan et al.^36^, who found that SCr was positively correlated with total hip BMD and lumbar spine BMD in subjects with normal renal function, and that 93.7% of the subjects included in this study had SCr values within the within normal levels, and only 6.3% of the subjects had abnormal SCr values with values > 133 μmol/L, which is consistent with the literature.

This study aimed to identify the risk factors for the occurrence of DOP and establish a nomogram to predict the risk rate for the same. Six predictors were screened and included in the nomogram. The predictive ability of the nomogram was 0.844 for risk prediction and 0.878 in the validation group. The advantages of this study mainly are four-fold: first, the use of the simple and objective clinical data to construct the prediction model; second, the variables used to construct the predictive model are easy to obtain, which greatly improves the model’s generalizability and facilitates its application to clinical practice; third, we firstly included indicators of malnutrition (GNRI) and explored their relationship with DOP; the last but not least, our nomogram showed a fair degree of differentiation, consistency, and calibration, the sensitivity was 0.883 and specificity was 0.643.

Several limitations should be considered. This is a single-center cross-sectional study with a limited sample size, which may introduce selection bias. In addition, serum hormones, bone metabolism indexes, and types of oral hypoglycemic agents used in patients with T2DM that may have a possible influence on the complication of DOP in elderly patients with T2DM were not included in the analysis of this study. The inclusion of factors that have an impact on DOP in the analysis is a limitation of this study. Only elderly T2DM patients were analyzed in this study, and further research is needed to determine whether this is applicable to other populations. Large-sample, multicenter, prospective studies can be conducted in subsequent studies to search for more risk factors for the complication of DOP in patients with T2DM so that relevant measures can be taken early to prevent the occurrence of DOP and delay the development of DOP.

## Conclusion

The present study demonstrated that gender, GNRI < 98, Ca, HbA1c, diabetes duration, and SCr are independent factors influencing the occurrence of DOP in elderly T2DM patients, and a dynamic nomogram was constructed to predict the risk of DOP. The result may guide clinical decision-making (Supplementary Information S1).

### Supplementary Information

Supplementary Information.

## Data Availability

All data generated or analyzed during this study are included in this published article and supplementary information files. Please contact the corresponding author for data requests.
